# Infrared Spectrum of Hydrobromic Aid

**DOI:** 10.6028/jres.064A.039

**Published:** 1960-10-01

**Authors:** Earle K. Plyler

## Abstract

A precise measurement of the infrared fundamental bands of HBr^79^ and HBr^81^ has been made. The two band centers have been found to be 2,558.94 and 2,558.56 cm^−1^, respectively. Rotational and vibrational constants have been calculated from the observed data. The constants are in good agreement with previous reported values. The centers of the two harmonic bands were used to calculate *ω_e_x_e_* and *ω_e_y_e_* and they were found to be 45.58 and 0.072 cm^−1^, respectively, for HBr^79^ and 45.56 and 0.072 cm^−1^ for HBr^81^.

## 1. Introduction

Hydrobromic acid was one of the first molecules whose spectrum was determined with a spectrometer of good resolution.^1^ The spectrum showed a series of lines which made up the *P*- and *R*-branches of the band. With improved instruments it was possible to partially resolve some of the lines of the fundamental band into two components.^2^ These components arise from the two isotopes of bromine and the separation is about 0.4 cm^−1^. Naudé and Verleger^3^ measured the 4–0 band of HBr in the photographic region and separated the isotopic bands. They calculated the rotational constants and their values will be compared with the results reported in this paper. More recently Thompson, Williams, and Callomon^4^ have measured the fundamental band with high resolution and have been able to separate the individual lines into two components. While the observed and calculated positions of the lines reported by them showed good agreement, it was thought that the absolute values of the line positions might be in error. A preliminary scan of the spectrum revealed that all the wavenumbers of the lines of HBr^79^ and HBr^81^ as measured by Thompson, Williams, and Callomon were too small by about 0.15 cm^−1^ and it was felt that a more precise measurement of the spectrum was needed so that this band could be used as a reference standard for calibration in the infrared region. Also, it was expected that a more accurate set of molecular constants could be obtained.

## 2. Experimental Results

The spectrum of HBr was observed on a high resolution spectrometer containing a grating with 10,000 lines/in. The ruled surface was 8¼ in. and was used double pass. A cool lead sulfide cell served as the detector and the resolution of the instrument was about 0.04 cm^−1^. The components of a line arising from HBr^79^ and HBr^81^ were completely separated. Further details of the instrument are given in a previous paper.^5^

The spectrum was measured by using the fringe system of a Fabry-Perot interferometer as a comparison spectrum. Reference standards were also superimposed on the recorded spectrum. Higher orders of the atomic lines of krypton were found to he well suited for the comparison spectrum.

A further method of insuring the absolute positions of the lines was employed. This method consisted of overlapping a section of the spectrum of HBr with that of the spectrum of HCl by putting both gases in the absorption cell, and then using the accurately known HCl lines as standards for the measurement of the HBr spectrum.

**Figure 1:**
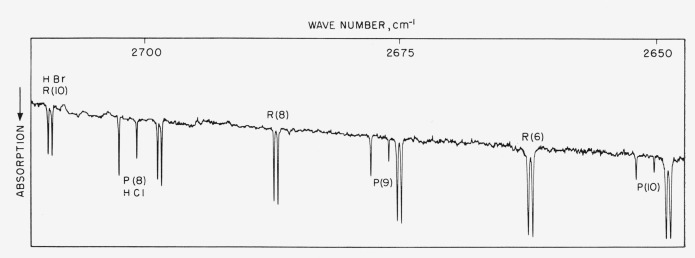
The absorption spectrum of *HBr* from R5 to R10 and *HCl* from P8 to P10. The pressure of each gas was 2.5 mm with a cell length of 6 m.

Figure 1 shows a section of the HBr spectrum from *R*5 through *R*10 overlapping the HCl spectrum from *P*8 to *P*10. The wavenumbers for the HBr lines from *R*5 to *R*10 as found by the two methods of measurement agreed to ±0.01 cm^−1^ and this result indicated that the absolute position of the band was well determined.

In order to have sufficient bands for determining the anharmonic terms *ω_e_x_e_* and *ω_e_y_e_* several lines of the first harmonic band were measured.

The calculated and observed positions of the rotational lines of the fundamental bands of HBr^79^ and HBr^81^ are listed in table 1. The values of the molecular constants were calculated and are listed in table 2. The following equation was used in determining the rotational constants:

$$\nu =\nu_{0}+(B_{1}+B_{0})m+(B_{1}-B_{0}-D_{1}+D_{0})m^{2}-(2D_{1}+2D_{0}-H_{1}-H_{0})m^{3}-[(D_{1}-D_{0})-3(H_{1}-H_{0})]m^{4}+3(H_{1}+H_{0})m^{5}$$ (1)

where *m*=*J*+1 for the *R*-branch and *−J* for the *P*-branch. The data of table 1 were reduced by means of an electronic computer.

The terms *ω_e_*, *ω_e_x_e_*, and *ω_e_y_e_* are found by using the folio wing equations:

ν1−0=ωe−2ωexe+314ωeye,2ν1−0−ν2−0=2ωexe−9ωeye,4ν1−0−ν4−0=12ωexe−78ωeye.} (2)

The observed wavenumbers of *ν*, 2*ν*, and 4*ν* and the calculated values of *ω_e_, ω_e_x_e_*, and *ω_e_y_e_* are given in table 3. The band centers for *ν* and 2*ν* have been determined in this work, but the value of 4*ν* has been taken from the work of Naudé and Verleger (see footnote 3, page 377).

The constants reported in table 2 are in good agreement with the results of Naudé and Verleger and those of Thompson, Williams, and Callomon, but the reported values differ more than the estimated accuracy given in the different reports. The greatest difference between the results reported in this paper and those of Thompson, Williams, and Callomon is the position of the band centers. They report *ν*_0_= 2,558.76 cm^−1^ for HBr^79^ and *ν*_0_=2,558.40 cm^−1^ for HBr^81^, while our measurements give the band centers at 2,558.94 cm^−1^ and 2,558.56, respectively. Also, the *B*_0_ values for the two molecules are greater by 0.0018 cm^−1^ in the measurements of Thompson, Williams, and Callomon than calculated from our measurements. It is believed that the molecular constants of HBr here presented are more precise than any previously reported. The probable error of the constants have been calculated and are included in table 2.

On the condition that the ratio of the molecular constants for HBr^79^ and HBr^81^ are only a function of the masses, the constants listed in table 2 for HBr^81^ have been calculated from the values of HBr^79^. The agreement between the molecular constants of HBr^81^ calculated from the measured lines listed in table 1 and the values calculated from HBr^79^ by the isotopic effect are all within the limit of the listed experimental error. This indicates that the relative values of the molecular constants for HBr^79^ and HBr^81^ are in excellent accord.

The error in the constants *D* and *H* is larger than in the *B* values. Only when a band has many lines which include large *J* values do these constants have a significant effect on a line position. That is, from eq (1) it is seen that the greatest contribution of *H* and *D* to the line position occurs in the terms *m*^3^, *m*^4^, and *m*^5^.

There are relationships between the molecular constants so that some of them can be calculated from the known values of others. For example, *D_e_* can be obtained from *B_e_* and *ω_e_* by the following relation:

$$D_{e}=4B_{e}^{3}/\omega_{e}^{2}.$$ (3)

From the above relation *D_e_*=3.459×10^−4^ cm^−1^ for HBr^79^ and *D_e_*=3.457×10^−4^ cm^−1^ for HBr^81^. These calculated constants are about two percent smaller than those obtained from the experimental data.

## Figures and Tables

**Table 1:** The observed and calculated positions of the lines of the fundamental bands of *HBr^79^* and *HBr^81^*

HBr^79^						
J	*R*_obs_	*P*_obs_	*R*_calc_	*P*_calc_	*R*_obs-calc_	*P*_obs-calc_
						
0	2,575.181	………………	2,575.176	………………	0.005	………………
1	2,590.945	2,542.240	2,590.939	2,542.237	.006	0.003
2	2,606.221	2,525.075	2,606.222	2,525.079	−.001	−.004
3	2,621.012	2,507.472	2,621.016	2,507.472	−.004	.000
4	2,635.314	2,489.423	2,635.314	2,489.427	.000	−.004
5	2,649.110	2,470.949	2,649.111	2,470.953	−.001	−.004
6	2,662.392	2,452.063	2,662.392	2,452.059	.000	.004
7	2,675.155	2,432.757	2,675.156	2,432.756	−.001	.001
8	2,687.393	2,413.060	2.687.395	2,413.055	−.002	.005
9	2,699.102	2,392.964	2,699.100	2,392.968	.002	−.004
10	2,710.266	………………	2,710.263	………………	.003	………………
11	2,720.876	………………	2,720.871	………………	.005	………………
12	2,730.928	………………	2,730.931	………………	−.003	………………
13	2,740.421	………………	2,740.420	………………	.001	………………
14	2,749.333	………………	2,749.333	………………	.000	………………

HBr^81^						
0	2,574.789	………………	2,574. 790	………………	−0.001	………………
1	2,590.555	2,541.865	2,590.549	2,541.862	.006	0.003
2	2,605.831	2,524.710	2,605.827	2,524.709	.004	.001
3	2,620.612	2,507.102	2,620.616	2,507.108	−.004	−.006
4	2,634.907	2,489.062	2,634.909	2,489.068	−.002	−.006
5	2,648.701	2,470.598	2,648.699	2.470.598	.002	.000
6	2,661.976	2,451.718	2,661.977	2,451.709	−.001	.009
7	2,674.732	2,432.408	2,674.737	2,432.411	−.005	−.003
8	2,686.969	2,412.717	2,686.972	2,412.714	−.003	.003
9	2.698.674	2.392.628	2,698.678	2,392.630	−.004	−.002
10	2,709.842	………………	2,709.844	………………	−.002	………………
11	2,720.448	………………	2,720.444	………………	.004	………………
12	2,730.494	………………	2,730.498	………………	−.004	………………
13	2,739.988	………………	2.739.989	………………	−.001	………………
14	2,748.907	………………	2,748.906	………………	.001	………………

**Table 2:** The calculated constants of *HBr^79^* and *HBr^81^* in cm^−1^

Constant	HBr^79^	HBr^81^
		
*ν*_o_	2,558.939 ± .001	2,558.559 ± .001
*B*_1_	8.1190 ± .0001	8.1165 ± .0002
*B_o_*	8.3516 ± .0001	8.3490 ± .0002
*D_1_*	3.37 ±.02 × 10^−4^	3.37 ±.02 × 10^−4^
*D_o_*	3.49 ± .02 × 10^−4^	3.48 ± .02 × 10^−4^
*H*	6.8 ±1 × 10^−8^	5.8 ± 1 × 10^−8^
*α*	0.2325 ± .00007	0.2325 ± .0001
*B_6_*	8.4678 ± .0001	8.4652 ± .0001
*B*	1.12 ±.11 × 10^−5^	1.10 ±.11× 10^−5^
*D_e_*	3.54 ±.02× 10^−4^	3.52 ±.02× 10^−4^

**Table 3:** The vibrational constants of *HBr^79^* and *HBr^81^* in cm^−1^

Constant	HBr^79^	HBr^81^
		
*ν*	2,558.939	2,558.559
2*ν*	5,027.378	5,026.636
4*ν*	9,694.495	9,693.151
ω*_e_*	2,649.855	2,644.450
ω*_e_x_e_*	45.576	45. 564
ω*_e_y_e_*	0.0724	0.0726

## Footnotes

1 H. M. Randall and E. S. Imes, Phys. Rev. 15, 152 (1920).

2 E. K. Plyler and E. F. Barker, Phys. Rev. 44, 984 (1933).

3 S. H. Naudé and H. Verleger, Proc. Phys. Soc. 63A, 470 (1950).

4 H. W. Thompson, R. L. Williams, and H. J. Callomon, Spectrochimica Acta 5, 313 (1952).

5 E. K. Plyler and L. R. Blaine, J. Research NBS 62, 7 (1959) RP2922.

